# Safety and Efficacy of Tumor-Treating Fields (TTFields) Therapy for Pediatric High-Grade Glioma: Results of a Prespecified Interim Analysis of the First Three Cases

**DOI:** 10.3390/children13010084

**Published:** 2026-01-06

**Authors:** Atsushi Makimoto, Keita Terashima, Ryo Nishikawa, Hiroyuki Fujisaki, Jun Kurihara, Satoshi Ihara, Jun-ichi Adachi, Mikako Enokizono, Naoko Mori, Yoshihiko Morikawa, Yuki Yuza

**Affiliations:** 1 Department of Transfusion and Laboratory Medicine, Tokyo Metropolitan Children’s Medical Center, 2-8-29, Musashidai, Fuchu, Tokyo 183-8561, Japan; 2 Department of Hematology/Oncology, Tokyo Metropolitan Children’s Medical Center, 2-8-29, Musashidai, Fuchu, Tokyo 183-8561, Japan; naoko_mori@tmhp.jp (N.M.); yuki_yuza@tmhp.jp (Y.Y.); 3 Clinical Research Support Center, Tokyo Metropolitan Children’s Medical Center, 2-8-29, Musashidai, Fuchu, Tokyo 183-8561, Japan; yoshihiko_morikawa@tmhp.jp; 4 Department of Neuro-Oncology, National Center for Child Health and Development, 2-10-1, Okura, Setagaya-ku, Tokyo 157-8535, Japan; terashima-k@ncchd.go.jp; 5 Department of Neuro-Oncology/Neurosurgery, Saitama Medical University International Medical Center, 1397-1, Yamane, Hidaka, Saitama 350-1298, Japan; rnishika@saitama-med.ac.jp (R.N.);; 6 Department of Pediatric Hematology/Oncology, Osaka City General Hospital, 2-13-22, Miyakojima-hondori, Miyakojima-ku, Osaka 534-0021, Japan; h-fujisaki@med.osakacity-hp.or.jp; 7 Department of Neurosurgery, Saitama Children’s Medical Center, 1-2, Shin-toshin, Chuo-ku, Saitama, Saitama 330-8777, Japan; kurihara.jun@saitama-pho.jp; 8 Department of Neurosurgery, Tokyo Metropolitan Children’s Medical Center, 2-8-29, Musashidai, Fuchu, Tokyo 183-8561, Japan; satoshi_ihara@tmhp.jp; 9 Department of Radiology, Tokyo Metropolitan Children’s Medical Center, 2-8-29, Musashidai, Fuchu, Tokyo 183-8561, Japan; mikako_enokizono@tmhp.jp

**Keywords:** tumor-treating fields therapy, pediatric high-grade glioma, glioblastoma, clinical trial, children

## Abstract

**Highlights:**

**What are the main findings?**

- Tumor-Treating Fields (TTFields) therapy was safe and feasible for pediatric diffuse high-grade glioma, the pediatric counterpart of adult glioblastoma.
- The efficacy of TTFields therapy needs to be assessed in detail after additional patient enrollment in this clinical study.

**What are the implications of the main findings?**

- Pediatric diffuse high-grade glioma is rare yet highly lethal, underscoring the urgent need for novel therapeutic approaches.
- This interim analysis supports further clinical development of TTFields therapy to accelerate regulatory approval for pediatric use.

**Abstract:**

**Background/Objectives**: Although Tumor-Treating Fields (TTFields) therapy is an established treatment modality for adult glioblastoma, clinical data on its efficacy in pediatric brain tumors are extremely scarce. The present study aimed to evaluate the safety of TTFields therapy for pediatric diffuse high-grade glioma (HGG) and to conduct an exploratory analysis of its efficacy. **Methods**: A prespecified, interim analysis was performed to determine whether the study should be continued on the basis of safety and feasibility data on the first three patients. The target population was children aged 5 to 17 years with newly diagnosed, supratentorial HGG or its first recurrence following frontline therapy. After completion of initial, local treatment for the tumor (surgical removal and/or radiotherapy), all patients received TTFields therapy using Optune^TM^ for 28 days per course for up to 26 courses until disease progression. **Results**: The interim analysis, which was completed in October 2022, included three female patients aged 14, 17, and 9 years. All had a histological grade 4 tumor, two of which were radiation-induced, secondary HGG. No serious, treatment-related toxicities or device-related issues were observed. All three patients were able to continue using the device for 75% or more of the time in accordance with the protocol, suggesting that the treatment was feasible. The MRI findings of two patients indicated that the treatment has a potential antitumor effect. Based on these results, the study was resumed and is currently being continued at multiple centers. **Conclusions**: The initial results of the prespecified, interim analysis demonstrated that TTFields therapy was safe and feasible for children with HGG. This study was funded by the Japan Agency for Medical Research and Development (AMED) and was registered with the Japan Registry of Clinical Trials (jRCTs032200423).

## 1. Introduction

Tumor-Treating Fields (TTFields) therapy is a novel treatment modality utilizing NovoTTF-100A or NovoTTF-200A (Optune^TM^, Novocure GmbH, Baar, Switzerland), which are medical devices that generate low intensity and intermediate frequency alternating electric fields [1,2]. It has been approved by the Food and Drug Administration (FDA) of the United States and its counterparts in other countries as a treatment for glioblastoma (GBM), one of the most aggressive types of malignant central nervous system (CNS) tumors in adults. TTFields therapy has been tested in multiple phase III trials and has shown promise in treating both recurrent [3] and primary GBM [4]. Several clinical guidelines consider TTFields therapy to be one of the standard treatments for adult GBM [5].

The Optune^TM^ system [6,7] consists of two primary components, an electric field generator and two pairs of transducer arrays, which are placed on opposite sides of the head with the tumor positioned directly between them to deliver the TTFields to the lesion non-invasively. The prevailing hypothesis holds that TTFields disrupt the localization and orientation of polar molecules related to mitotic microtubules, including tubulin and septin, thereby inhibiting tumor cell proliferation and inducing tumor cell death [1,2]. In addition, TTFields may also inhibit DNA damage repair [8], impair cellular migration and invasion [9], upregulate autophagy [10], enhance tumor immunity [11], etc.

Pediatric diffuse high-grade glioma (pediatric HGG), newly defined in the latest World Health Organization (WHO) classification of CNS tumors [12], was previously regarded as the counterpart of adult GBM [13]. The physical mechanism of TTFields therapy and similarities between pediatric HGG and adult GBM, such as the dismal prognosis, pathological features, and treatment response pattern, may justify the application of TTFields therapy to pediatric HGG. However, the safety, feasibility, and efficacy of TTFields therapy for children await rigorous testing in a clinical trial.

The present clinical study was conducted to evaluate the safety, feasibility, and potential efficacy of Optune^TM^ as a treatment for pediatric HGG with the ultimate aim of overcoming the regulatory obstacle preventing expansion of the indications of Optune^TM^ to include pediatric HGG. We herein present the results of a prespecified interim analysis of the first three enrolled patients.

## 2. Materials and Methods

### 2.1. Study Design

The present, open-label, phase-2 type, multicentric study was designed to evaluate the safety and feasibility of second-generation Optune^TM^ [7] for the treatment of pediatric HGG and to conduct an exploratory analysis of its efficacy. The study commenced at a single institution (Tokyo Metropolitan Children’s Medical Center) and continued there until the first three patients were enrolled. When the third patient became evaluable for safety and feasibility assessment of the first course of the treatment, patient enrollment was halted, and safety and feasibility data were collected for an interim analysis. If the results justify continuing the study, registration will resume as planned to include up to ten patients drawn from multiple centers. Study enrollment began in April 2021 and continued through September 2024. Follow-up observation will end in September 2026.

### 2.2. Patient Cohort

Patients aged between 5 and 17 years with pediatric HGG originating in the supratentorial region were considered suitable for enrollment. The updated 4th edition of the WHO classification of CNS tumors [13] was initially used to classify the tumors. Following a revision of the study protocol, the 5th edition of the same guidelines [12] was adopted in May 2022. Throughout this transition period, only histological grade 4 tumors, including therapy-related, secondary tumors, were considered eligible. The eligibility criteria included newly diagnosed disease, the first recurrence, Karnofsky/Lansky performance status ≥ 70, and written informed consent from the patients and/or their legal guardian. Patients were excluded if they had a life expectancy < 3 months, a serious, uncontrollable infection or organ dysfunction as complications, an interval > 100 days from the confirmatory diagnosis, implantation of a medical device, implantation of an intraventricular shunt tube or pregnancy.

### 2.3. Treatment

Briefly, the patients receiving TTFields therapy using second-generation NovoTTF-100A (Optune^TM^, Novocure GmbH, Baar, Switzerland) had four transducer arrays placed on their shaved scalp and connected to a portable device set to generate electric fields in the brain with a 200 kHz frequency and 1 V/cm intensity. The device was operated in accordance with instructions for use and the user manual provided by Novocure. The two pairs of disposable transducer arrays were replaced two to three times per week. Because continuous contact with the scalp frequently caused skin-related adverse events such as redness, erosion, ulceration, and contact dermatitis, the patients and their guardians were instructed to take prophylactic measures, including proper shaving, appropriate scalp hygiene, and relocating the arrays.

TTFields therapy began after completing appropriate, local treatment of the tumor (surgical removal and/or radiotherapy as appropriate), and was continued for 28 days per course for up to 26 courses until the end-of-therapy criteria, including tumor progression, were met. Concomitant treatment with chemotherapy was allowed if it was part of the standard therapy for pediatric HGG.

### 2.4. Evaluation and Endpoints

The primary endpoint was the rate of adverse events with causality. The secondary endpoints included the response rate, clinical benefit rate, progression-free survival (PFS) rate, overall survival (OS) rate, quality of life (QoL), and the adverse event rate. Adverse events were assessed using the Common Toxicity Criteria for Adverse Events, ver. 4.0 [14]. Magnetic resonance imaging (MRI) will be performed every two months to assess the treatment’s efficacy using McDonald’s [15] and the RANO-HGG [16] criteria. QoL was evaluated every three months using PedsQL^TM^ (Mapi Research Trust, Lyon, France), which includes both the Generic Core scale [17] and the Brain Tumor Module [18].

### 2.5. Evaluations in the Interim Analysis

**Figure 1:**
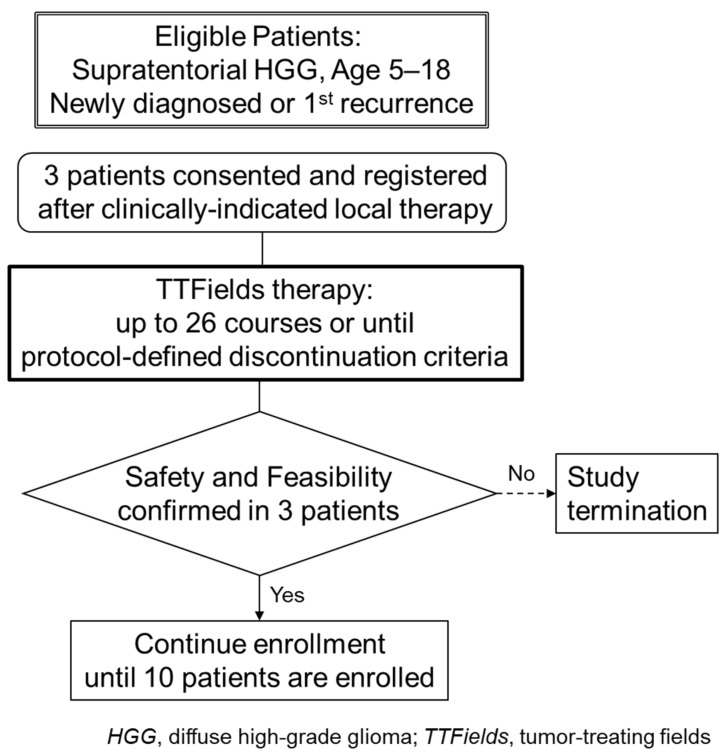
Flow diagram of the study.

The interim analysis evaluated early-stage safety and feasibility of the treatment in the first three enrollees (Figure 1). The absence of any serious, adverse reactions, defined as adverse events with a causal relationship or device deficiencies causing serious outcomes, was treated as evidence of early-stage safety. The treatment was considered feasible if it was able to be administered for 18 h/day on average [19] in all three patients. An exploratory analysis of the QoL data was performed to supplement the results of the feasibility assessment. This interim analysis report was written essentially following the TREND guidelines.

### 2.6. Ethics Approval for the Study

The present study was approved by the Clinical Research Review Board of Tokyo Metropolitan Geriatric Medical Center (certified by the Ministry of Health, Labour and Welfare of Japan [MHLW]) in December 2020 (protocol code: H20-04). The study was begun under the auspices of the Advanced Medical Care Program [20] and with the approval of the MHLW from April 2021. The study was registered with the Japan Registry of Clinical Trials (jRCT) on 16 March 2021, with the registration code of jRCTs032200423 (https://jrct.mhlw.go.jp/latest-detail/jRCTs032200423 [accessed on 18 September 2025]).

Informed consent and assent (when appropriate) were obtained from the patients and their guardians in accordance with local institutional guidelines, the Clinical Trial Act of Japan, and the Declaration of Helsinki. An independent data-monitoring committee (DMC) continuously monitored the progress and safety of the study.

## 3. Results

### 3.1. Patient Background

Table 1 summarizes the background of the three patients. Two patients had radiation-induced glioma (RIG) [21], and one patient had glioblastoma classified as *IDH*-wildtype by the updated 4th edition of the WHO classification [13]. None of the patients had a comorbidity. The first patient was registered in November 2021. The planned interim analysis was performed in October 2022 after the third patient completed the first course of TTFields therapy.

### 3.2. Safety

All three patients experienced eight adverse events in total, which were related to the therapy (Table 2). Six events were common, skin-related adverse events. The remaining two events (a fall and hyperventilation) occurred in the patient in Case 1, who experienced rapid disease progression after entering the study as described below. Deterioration of the patient’s condition probably contributed to the occurrence of these events. No serious adverse event related to TTFields therapy was noted. The failure of portable batteries in Case 2 was the only instance of device failure and did not constitute a serious event.

### 3.3. Feasibility

The feasibility criterion was the continued use of the device for 75% or more of the allotted time (18 h/day). Only the patient in Case 1 failed to meet this criterion during course #1 for the case-specific reason described in the individual clinical summary below. After the array layout was changed, the patient was able to meet the criterion (i.e., the hours of use increased to 76%). The remaining two patients met the criterion in all the courses evaluated in this analysis (on average 93% and 87% in Case 2 and Case 3, respectively).

### 3.4. Quality of Life

**Figure 2:**
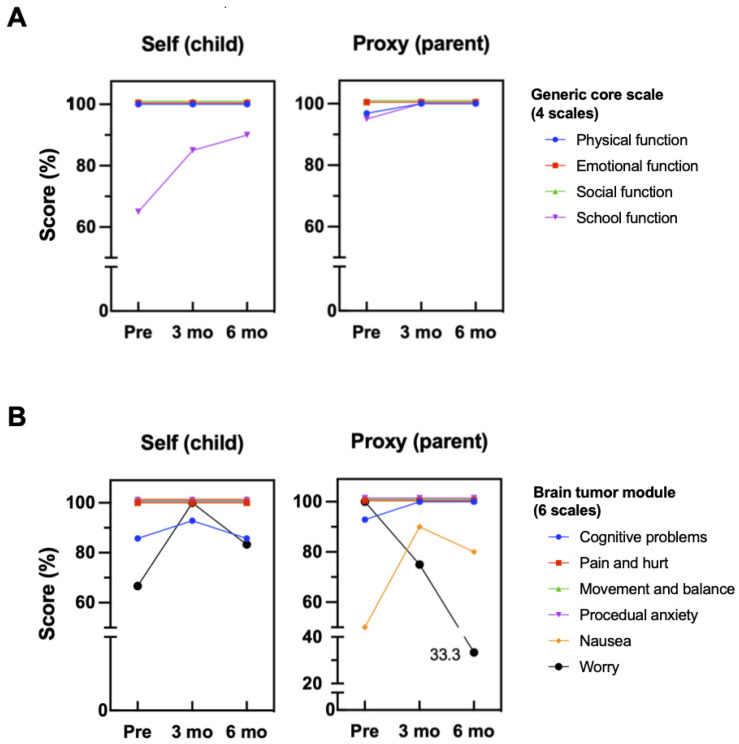
Time-series changes in the quality of life (QoL) scores using the PedsQL^TM^ generic core scale (**A**) and the brain tumor module (**B**) in Case 2, showing a good response to the therapy in all the categories except “worry”.

Analysis of the QoL data from two patients was difficult because the patient in Case 1 received only two courses, and the patient in Case 3 had just finished the first course of the TTFields therapy before the start of the interim analysis. Only the patient in Case 2 had all the data for the three points (pre-treatment, month 3, and month 6). Figure 2 summarizes the time series change in the score per category in Case 2. Both the self-rating and the proxy-rating, except for the score for “worry,” demonstrated a good response to the therapy. The score for “cognitive problems” and “anxiety” on the Brain Tumor Module and “physical functioning” and “school functioning” on the Generic Core Scale continued to improve during the treatment.

### 3.5. Clinical Summary of Representative Case

Because the patient in Case 3 had just completed the first course of TTFields therapy at the time of the interim analysis, a detailed clinical summary of only two remaining cases is presented below.

#### 3.5.1. Case 1

Case 1 involved a 14-year-old female patient who had originally been diagnosed with acute lymphoblastic leukemia at the age of six and had experienced repeated bone marrow and central nervous system relapses. After achieving a second remission with multi-agent chemotherapy, she underwent an unrelated donor bone marrow transplantation (BMT) following a conditioning regimen consisting of 12 Gy total body irradiation, 6 Gy craniospinal irradiation, and melphalan at 180 mg/m^2^, which resulted in a third remission. RIG originating in the left temporal lobe developed 65 months after the BMT. She underwent gross total resection of the tumor followed by chemoradiotherapy (54 Gy XRT plus temozolomide) but experienced a local recurrence seven months later during maintenance therapy using temozolomide. She was enrolled in the present study following her first recurrence, which was treated with a gross total resection and radiation therapy (30.6 Gy). Disseminated progression occurred just after enrollment, and TTFields therapy began with the permission of the independent DMC. Although a special “posterior-tentorial array layout plan” was applied to cover the posterior-fossa lesions [22], the electrodes could not be fastened firmly to the posterior neck because of a steroid-induced buffalo hump. The treatment became feasible after switching to the generic layout. TTFields therapy was terminated due to disease progression during the second course of treatment. The patient died from disease progression complicated by sepsis.

**Figure 3:**
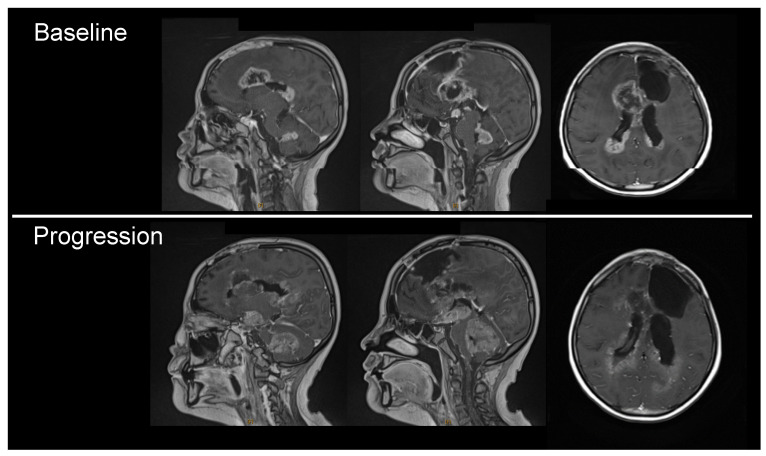
Contrasting progression patterns in the supra- and infra-tentorial lesions in Case 1 (Gd-enhanced T1-weighing image) suggesting a discernible treatment effect on the supra-tentorial lesions.

MRI demonstrated contrasting progression patterns in the supra- and infra-tentorial lesions, suggesting that the TTFields therapy had some anti-tumor effect (Figure 3). Specifically, MRI demonstrated multiple lesions mainly in five areas, including the right frontal lobe, genu corporis callosi, bilateral lateral ventricles, hypothalamus, and cerebellum. Although the overall outcome was progressive disease (PD), two of the lesions in the supra-tentorial area (the right frontal lobe and genu corporis callosi) demonstrated signs of continued shrinkage at the last evaluation; the sum of the product of the perpendicular diameters of these two lesions had decreased from 1976 mm^2^ to 1458 mm^2^. In contrast, the sum of the product of the perpendicular diameters of the remaining three lesions had markedly increased from 656 mm^2^ to 2284 mm^2^.

#### 3.5.2. Case 2

**Figure 4:**
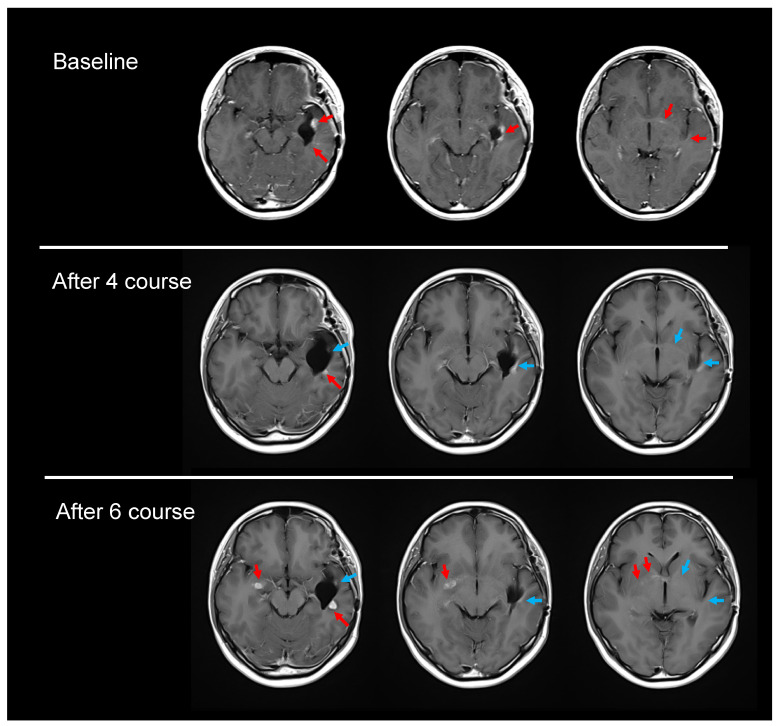
Temporary partial and mixed responses in Case 2 (Gadolinium-enhanced T1-weighing imaging). The red arrows indicate stable, progressive lesions, and the blue arrows indicate shrinking lesions.

Case 2 involved a 17-year-old female patient with a glioblastoma in the left temporal lobe. She had undergone a subtotal resection of the tumor and 60 Gy XRT with concurrent temozolomide administration. She was immediately enrolled in the present study and began receiving TTFields therapy. MRI demonstrated improvement until the end of the fourth course of treatment. Progressive disease then developed, and the therapy was discontinued after completion of the sixth course. Major measurable lesions occurred in the post-surgical cavity in the left lateral lobe. Sequential MRI revealed that the sum of the product of the perpendicular diameter of these lesions, which was 90 mm^2^ at the baseline, decreased to 40 mm^2^ by the end of the fourth course, suggesting a partial response (PR). However, this change did not last long enough to be confirmed, as the appearance of new, contralateral lesions two months later (Figure 4) indicated PD. With discontinuation of TTFields therapy following completion of the sixth course, she received pembrolizumab 200 mg because the tumor tissue demonstrated high-grade microsatellite instability. However, her disease rapidly progressed after the first dose of pembrolizumab, and she died more than two months (64 days) after the termination of the TTFields therapy, suggesting that the TTFields therapy might have had a tumor-suppressive effect.

## 4. Discussion

Pediatric GBM/HGG is rare yet highly lethal [23], making the development of a novel treatment extremely challenging but absolutely necessary. According to the Central Brain Tumor Registry of the United States (CBTRUS), GBM accounts for 14.2% of all primary CNS tumors (n = 453,623), although the proportion decreases to 2.6% in children and adolescents aged 0–19 years (n = 24,999) [24].

Although pediatric GBM was subsumed to pediatric HGG in the 2021 WHO classification, grade 4 pediatric HGG is still regarded as the clinical counterpart of adult GBM. There are age-specific tumor genotypes, such as H3K27M and H3G34R, in pediatric HGG, but the phenotype, including the microscopic morphology and clinical tumor behavior, is common to adult GBM and pediatric HGG. Because the effectiveness of TTFields therapy depends on an interaction between its physiological activity and tumor cell division, differences in the tumor genotype should not greatly influence the effectiveness of this therapy.

Branter et al. [25] assessed the effect of TTFields on brain tumor cell lines, including GBM, medulloblastoma, and ependymoma. In their study, a panel of tumor cell lines was treated with a range of clinically relevant frequencies (100–400 kHz) for 72 h. TTFields therapy achieved a significant reduction in the number of cells in both pediatric and adult cell lines in a voltage- and frequency-dependent manner [25]. These *in vitro* findings also supported the extrapolation of TTFields therapy from adult GBM to its pediatric counterpart.

The present three cases demonstrated that the treatment had a good safety profile and feasibility. In Case 1, the patient experienced a rapid deterioration of her condition together with several adverse events related to the disease. However, even in this serious case, TTFields therapy had an acceptable level of safety and feasibility. The patients in Cases 2 and 3 experienced adverse events affecting only the scalp.

There are three reports of global, post-marketing safety surveillance, which include all the currently available data on TTFields therapy in pediatric patients [26,27,28]. Although some of these data were redundant, none of the studies reported any issues with the safety of the therapy when administered to children. A study by Mrugala et al. [28], which enrolled the largest cohort to date of 25,898 patients with GBM and other brain tumors of whom 93 (0.4%) were pediatric patients aged less than 18 years, found a similar incidence and patterns of therapy-related adverse events in the pediatric and adult subpopulations. No unexpected toxicities were identified in the pediatric population in any of the studies.

As of August 2025, there are only four studies describing clinical data on TTFields therapy in pediatric patients [29,30,31,32]. These studies collectively included 11 patients aged 3–20 years (median: 13 years) with GBM (n = 5), gliomatosis cerebri (GC, n = 2), diffuse midline glioma (DMG) with a H3K27M variant (n = 2), RIG (n = 1), and anaplastic oligodendroglioma (n = 1). Two cases of scalp irritation occurred, but no safety problems were reported.

In terms of feasibility, the three patients in the present study achieved an acceptable level of adherence to the therapy. In the four studies cited above, a report on treatment adherence was available for five of the 11 patients. Three patients, including two toddlers, achieved an acceptable level of treatment adherence [29,31,32].

Taken together, TTFields therapy using the same device as in adults appeared to be safe, feasible, and applicable to pediatric patients with a brain malignancy.

The paucity of data on the efficacy of TTFields therapy rendered an objective analysis impossible. In the present study, the patient in Case 1 demonstrated contrasting progression patterns in the supra- and infra-tentorial lesions, suggesting that the therapy had some antitumor effect on the supra-tentorial lesions (Figure 3). Glas et al. [33] reported a direct correlation between TTFields dose distribution and the tumor response, which is supported by the paradoxical response involving the supra- and infra-tentorial lesions in Case 1.

The patient in Case 2 achieved transient PR on MRI until the end of the fourth course, but objective progression became evident at the end of the sixth course, when the patient discontinued TTFields therapy in accordance with the protocol. Afterwards, the disease showed rapid progression, which was later accompanied by a marked decline in the patient’s clinical condition. The patient died approximately two months later, suggesting that TTFields therapy was still controlling the disease even after the onset of tumor progression.

In the four studies cited earlier (n = 11), three patients with GBM, GC, and DMG, respectively, achieved PR, and two with GBM and DMG, respectively, achieved stable disease status over six months. Although the survival benefit is objectively unevaluable, TTFields therapy has the potential to exert an antitumor effect on at least some children with HGG, including GBM, GC, and DMG.

Wearing a transducer array all day will clearly negatively impact the patient’s QoL. Thus, analyzing QoL data pertaining to TTFields therapy is very important. In the present study, the QoL score of the patient in Case 2 demonstrated good overall tolerance, and most of the scores, such as that for ‘cognitive problems,’ ‘anxiety,’ and ‘school functioning,’ continued to improve during the treatment. The pattern of improvement in the QoL scores was comparable to that seen in adult GBM patients who received TTFields therapy, suggesting that the improvement in the QoL score is likely to be related to the therapeutic effect [34,35].

In the present study, close attention was paid to three differences between applying the treatment to pediatric patients and applying it to adult patients: (1) Using appropriate explanatory documents and assent forms, a thorough explanation about the treatment and its importance was provided not only to the patients’ guardians but also to the patients themselves before obtaining their assent. (2) Teachers of school-aged children were also given a thorough explanation about the treatment and the device used before requesting their cooperation in managing possible issues, such as device malfunction and charging the battery. (3) In view of children’s high activity levels, families were asked to schedule treatment-free periods during physical activities or leisure time to allow the patients to continue enjoying their daily life. These measures contributed to maintaining the highest possible quality of life for all three of the patients in this study.

The goal of the current study is to overcome the regulatory barriers to expanding the indications for second-generation Optune^TM^ to include pediatric HGG in Japan, where the off-label use of medical devices is impossible owing to the lack of coverage by the national health insurance system [36]. Therefore, at least a partial change in the regulatory status and labeling of Optune^TM^ would be desirable as a first step toward this goal. Discussions with the Pharmaceuticals and Medical Devices Agency (PMDA), the regulatory agency in Japan, have led to a tentative consensus that the accumulated data on the efficacy of Optune^TM^ for adult GBM may be extrapolatable to pediatric HGG if the present study succeeds in demonstrating a level of efficacy equivalent to that reported in adults. At the same time, the accumulation of pediatric safety data in the current study, other clinical studies, and post-marketing surveillance studies may expedite the approval of the device for use as a treatment for pediatric HGG.

As of August 2025, there are two clinical trials of pediatric TTF therapy, both of which are underway in the USA (ClinicalTrials.gov). One is the HUMC 1612 trial (NCT03128047) [37], a bicentric, open-label, uncontrolled trial examining the safety of Novo-TTF-200A combined with temozolomide and bevacizumab administration during 56 days of treatment in children with high-grade malignant glioma. The other is the Pediatric Brain Tumor Consortium (PBTC) -048 trial (NCT03033992) [38], a multicentric, open-label, uncontrolled trial enrolling patients aged 5–21 years with recurrent or refractory malignant glioma or ependymoma and having the primary objectives of establishing the feasibility of the treatment and collecting toxicity data. PBTC-048 has opened a second stratum involving concomitant radiotherapy and maintenance TTFields therapy for children with DIPG. The results of the two trials, which have yet to be published, should contribute greatly to achieving the goal of the present study of obtaining regulatory approval.

One limitation of the present study is the small number of participants and the availability only of the results of the interim analysis of the first three patients with pediatric HGG. However, even at the current stage, the data demonstrated good safety and feasibility of the treatment in children. Our study’s assessment of the safety and feasibility of TTFields therapy needs to be completed in order to obtain regulatory approval for the expansion of the indications for Optune^TM^ to include pediatric HGG. Patient enrollment was completed by September 2024, and the enrollees are currently being followed. The final analysis will be conducted at the conclusion of an observation period, which is expected to last no more than two years.

## 5. Conclusions

TTFields therapy demonstrated adequate safety and feasibility in three prospectively registered pediatric patients with HGG, thus warranting the continuation of the present study. International collaboration aimed at sharing the treatment outcomes of TTFields therapy for pediatric HGG and other brain tumors is desirable to justify the expansion of its clinical indications to include pediatric patients.

## Figures and Tables

**Table 1:** Patient characteristics.

PIN	Age/Sex	Diagnosis	Histology	Site	Disease status
1	14/F	Radiation-induced glioma	Grade 4	Lt. frontal lobe	1st recurrence
2	17/F	Glioblastoma, *IDH*-wildtype	Grade 4	Lt. temporal lobe	Newly diagnosed
3	9/F	Radiation-induced glioma	Grade 4	Bil. ant. horns of lateral ventricles	Newly diagnosed

ant., anterior; Bil., bilateral; F, female; *IDH*, isocitrate dehydrogenase (gene); Lt., left; PIN, patient identification number.

**Table 2:** Adverse Events and Device Failure.

PIN	Course#	Adverse Events/Device Failure	Grade
1	1	Skin erosion	2
1	1	Scalp skin redness	1
1	1	Fall	2
1	1	Hyperventilation (psychiatric disorder)	1
1	1	Skin ulceration	2
2	1	Scalp skin redness	2
3	1	Skin indentation	1
3	1	Contact dermatitis	1

PIN, patient identification number.

## Data Availability

The data generated and/or analyzed during the current study are available from the corresponding author on reasonable request.

## Abbreviations

The following abbreviations are used in this manuscript:

TTFs	Tumor-treating fields
HGG	High-grade glioma
FDA	Food and Drug Administration
GBM	Glioblastoma multiforme
CNS	Central nervous system
DNA	Deoxyribonucleic acid
WHO	World Health Organization
TM	Trademark
PFS	Progression-free survival
OS	Overall survival
QoL	Quality of life
MRI	Magnetic resonance imaging
RANO	Response Assessment in Neuro-Oncology
MHLW	Ministry of Health Labour and Welfare
DMC	Data monitoring committee
RIG	Radiation-induced glioma
IDH	Isocitrate dehydrogenase
XRT	X-ray radiotherapy
mo	Months
Gy	Gray
CBTRUS	Central Brain Tumor Registry of the United States
GC	Gliomatosis cerebri
DMG	Diffuse midline glioma
PMDA	Pharmaceutics and Medical Devices Agency
USA	United States of America
HUMC	Hackensack University Medical Center
PBTC	Pediatric Brain Tumor Consortium
